# Type 1 Diabetes at-risk children highly recognize *Mycobacterium avium* subspecies *paratuberculosis* epitopes homologous to human Znt8 and Proinsulin

**DOI:** 10.1038/srep22266

**Published:** 2016-02-29

**Authors:** Magdalena Niegowska, Novella Rapini, Simona Piccinini, Giuseppe Mameli, Elisa Caggiu, Maria Luisa Manca Bitti, Leonardo A. Sechi

**Affiliations:** 1 Università degli Studi di Sassari, Dipartimento di Scienze Biomediche, Sezione di Microbiologia e Virologia, 07100 Sassari, Italy; 2 Pediatric Diabetology Unit, Policlinico di Tor Vergata, University of Rome Tor Vergata, 00133 Rome, Italy

## Abstract

*Mycobacterium avium* subspecies *paratuberculosis* (MAP) has been previously associated to T1D as a putative environmental agent triggering or accelerating the disease in Sardinian and Italian populations. Our aim was to investigate the role of MAP in T1D development by evaluating levels of antibodies directed against MAP epitopes and their human homologs corresponding to ZnT8 and proinsulin (PI) in 54 T1D at-risk children from mainland Italy and 42 healthy controls (HCs). A higher prevalence was detected for MAP/ZnT8 pairs (62,96% T1D vs. 7,14% HCs; p < 0.0001) compared to MAP/PI epitopes (22,22% T1D vs. 9,52% HCs) and decreasing trends were observed upon time-point analyses for most peptides. Similarly, classical ZnT8 Abs and GADA decreased in a time-dependent manner, whereas IAA titers increased by 12%. Responses in 0–9 year-old children were stronger than in 10–18 age group (75% vs. 69,1%; p < 0.04). Younger age, female sex and concomitant autoimmune disorders contributed to a stronger seroreactivity suggesting a possible implication of MAP in multiple autoimmune syndrome. Cross-reactivity of the homologous epitopes was reflected by a high correlation coefficient (r^2^ > 0.8) and a pairwise overlap of positivity (>83% for MAP/ZnT8).

Even though type 1 diabetes (T1D) is one of the most common autoimmune diseases with a several-fold increase over the last decades, knowledge regarding the factors contributing to its development remains as yet incomplete. The genetic background has been considered as a major contributor until twin and migration studies along with in-depth association analyses involving HLA genotypes showed only partial concordance, suggesting a combination of factors with a strong environmental impact on disease progression. Production of antibodies (Abs) to pancreatic islet cells even before recognition of clinical symptoms provides a primary diagnostic target to define T1D onset in at-risk subjects. In the last years, analyses of classical islet autoantibodies have been supported by detection of Abs against the β-cell antigen zinc transporter 8 (ZnT8) through a test engaging a fusion protein that combines extra-luminal regions^1^.

*Mycobacterium avium* subspecies *paratuberculosis* (MAP) has been previously associated to T1D as a putative environmental agent triggering or accelerating the disease in Sardinian and Italian populations^2^,^3^,^4^,^5^,^6^. This hypothesis is based on a few findings. First, MAP causes Johne’s disease in ruminant livestock worldwide and is shed into milk of infected animals even during the asymptomatic phase. It’s presence has been detected in dairy products comprising infant formulas^7^,^8^,^9^,^10^, thus risk of transmission may be enhanced since early childhood. Second, we have documented that Abs directed against MAP-derived peptides present high prevalence among T1D patients (up to 55,2%) when compared to healthy volunteers (6,7%)^11^. The MAP peptides identified within different proteins (MAP2404c, MAP1,4-α-glucan branching protein and MAP3865c) are characterized by sequence homology with proinsulin (PI) and ZnT8 transmembrane domain; the outcomes of competitivity assays clearly demonstrate their cross-reactivity^12^,^13^ and allow to hypothesize the role of molecular mimicry through which MAP contribution to T1D development may occur. Finally, we have isolated MAP DNA from 63% of the analyzed T1D Sardinian patients^2^ and cultured intact bacilli from blood^3^.

Our recent study investigated Abs levels during the prediabetes period in a small Sardinian cohort^14^ revealing that anti-MAP and anti-ZnT8/PI Abs frequently appear right after birth preceding the first classical ZnT8 and insulin autoantibodies undetectable before six months of age^15^. What is more, most subjects who progressed to diabetes were reactive to MAP and the homolgous human peptides. As Sardinian populations display a high genetic homogeneity stemming from the shared ancestry coupled with evolutionary forces^16^ and resulting in susceptibility to autoimmune diabetes, our objective was to evaluate responses against the same peptide selection in subjects from a different biogeographical background. Considering the estimates that MAP infections among cattle herds in Sardinia are of particularly high frequency reaching 60%^17^, exposure to MAP of an external population would occur with minor intensity providing an important ground for comparison of the two cohorts.

In the present study, we investigated whether the Abs pattern involving sero-reactivity to MAP-derived peptides and their human ZnT8/PI homologs in children and youth at risk for T1D from mainland Italy reflects prevalences reported for new-onset T1D Sardinian and Italian pediatric patients^4^,^11^. In order to evaluate a possible role of anti-MAP Abs as early predictive biomarkers, we performed a correlation with classical islet autoantibodies. Moreover, we analyzed time-point variations in Abs positivity with reference to the further onset of T1D and risk factors including HLA genotype, concomitant diseases and familiarity.

## Results

### Prevalence of Abs against MAP/ZnT8 and MAP/PI homologus epitopes in T1D at-risk subjects and age-matched HCs

Among 54 subjects at risk of T1D, 70,37% (n = 38) resulted positive to at least one of the eight assessed peptides compared to 16,67% (n = 7) of HCs. 78,95% (n = 30) of the positive at-risk children had Abs targeting not less than four epitopes, 11,11% of whom were fully responsive to all peptide pairs. Considering healthy controls, multiple serum reacivity to at least three peptides was observed in four volunteers, whereas the remaining three positive subjects responded only to PI_46–61_, PI_64–80_ or MAP3865c_133–141_.

Upon single-peptide analysis of Abs reactivity, MAP3865c_133–141_ was recognized by 61,11% of at-risk subjects and by only 7,14% of HCs (AUC = 0.72; p < 0.0001). The highest immunoreactivity among at-risk children was detected for ZnT8_186–194_ and reached 62,96% in comparison to 7,14% among HCs (AUC = 0.74; p < 0.0001).

Serum Abs reactivity to MAP3865c_125–133_ accounted for 62,96% as well (AUC = 0.80; p < 0.0001), while recognition of the homologous ZnT8_178–186_ was observed in 51,85% (AUC = 0.75; p < 0.0001) of at-risk subjects, comparing to 4,76% of HCs for both peptides.

Concerning MAP homolgs of proinsulin-derived peptides, anti-MAP1,4αgbp_157–173_ Abs were found in 22,22% of at-risk children and in 7,14% of HCs (AUC = 0.58; p < 0.17). Abs positivity against PI_64–80_ accounted for 16,67% among at-risk individuals, however prevalence among HCs maintained levels of 7,14% detected for the homologous peptide (AUC = 0.57; p < 0.19).

**Figure 1:**
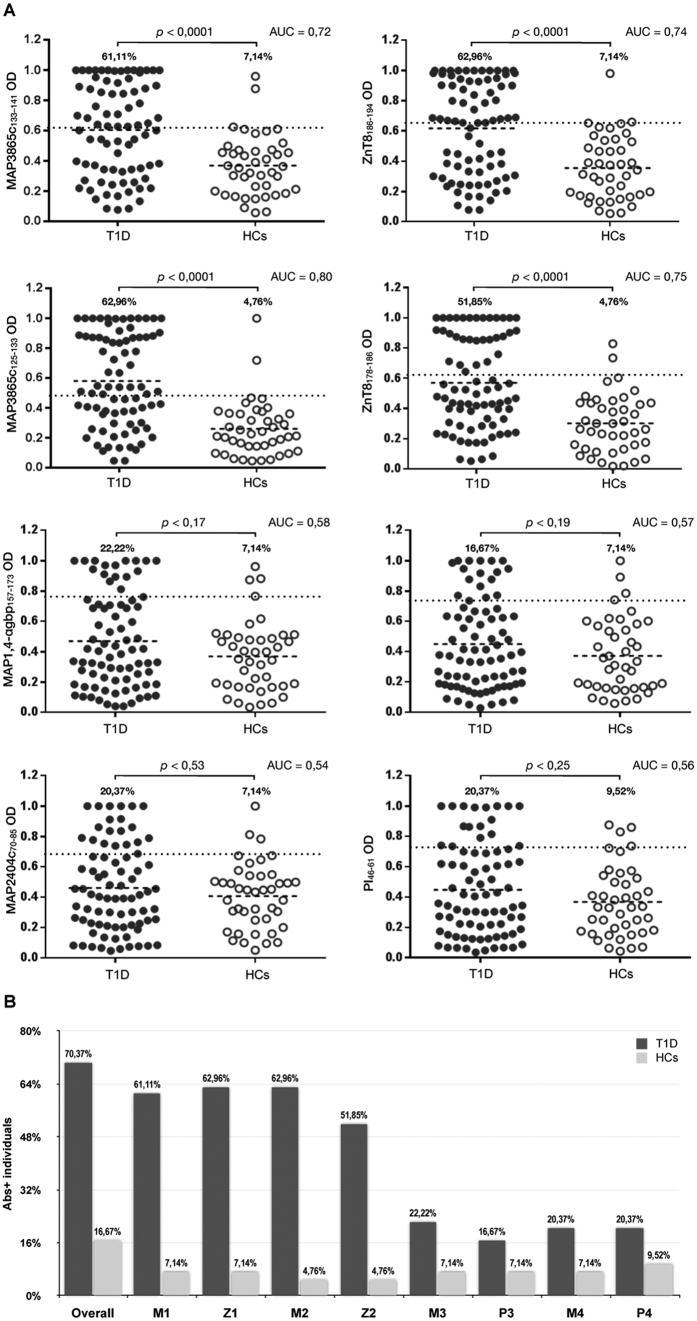
Prevalence of Abs against MAP, proinsulin and ZnT8 homologous epitopes in T1D at-risk subjects and healthy controls. Sera were tested in duplicate for their reactivity against plate-coated peptides: MAP3865c_133–141_ (M1); ZnT8_186–194_ (Z1); MAP3865c_125–133_ (M2); ZnT8_178–186_ (Z2), MAP1,4αgbp_157–173_ (M3); PI_64–80_ (P3); MAP2404c_70–85_ (M4); PI_46–61_ (P4). (**A**) Distribution of Abs values based on the statistical analyses performed for all peptides separately. The dotted lines indicate thresholds of positivity relative to each assay calculated by ROC analysis. The percentage of Abs-positive at-risk subjects is reported on top of each distribution; horizontal bars specific for T1D and HCs groups correspond to means. AUC and p values (CI 95%) are indicated above the graphs. (**B**) Percentage of children with Abs positivity to selected epitopes upon single-peptide analysis. The first column pair summarizes reactivity to any of the analysed peptides. Dark bars represent subjects at risk for T1D; light grey bars correspond to HCs.

20,37% of at-risk subjects reacted to both MAP2404c_70–85_ and PI_46–61_ compared to 7,14% (AUC = 0.54; p < 0.53) and 9,52% (AUC = 0.56; p < 0.25) of positivity to the respective epitopes found among HCs. Figure 1 depicts percentages of each peptide pair. Interestingly, a slightly higher prevalence of positivity to MAP/PI peptides compared to MAP/ZnT8 was observed among HCs, in contrast to at-risk subjects presenting an inverse picture.

**Figure 2:**
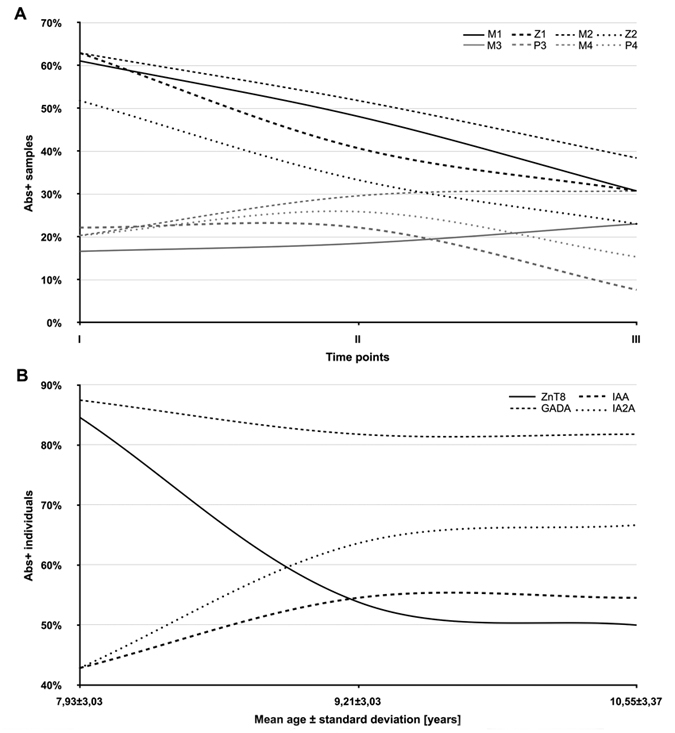
Time-related prevalence of Abs directed against the homologous epitopes and classical islet autoantibodies in children at risk for T1D. (**A**) Three time-point variations in Abs reactivity to the following peptides were analyzed: MAP3865c_133–141_ (M1); ZnT8_186–194_ (Z1); MAP3865c_125–133_ (M2); ZnT8_178–186_ (Z2), MAP1,4αgbp_157–173_ (M3); PI_64–80_ (P3); MAP2404c_70–85_ (M4); PI_46–61_ (P4). MAP/ZnT8 homologs are indicated by black lines, while gey lines indicate MAP/PI homologous epitopes. (**B**) Time-dependent classical Abs status evaluated including ZnT8, GADA, IAA and IA2A in 13 at-risk subjects.

When time-point samples collected within further four years were analyzed for the presence of Abs against the homologous peptides, sero-reactivity appeared maintained in 2 out of 3 at-risk subjects initially positive for the full set of epitope pairs. Similarly, Abs-negative status was constant in children (n = 3) not demonstrating any response at the first blood collection. Fluctuations involving responses to the selected peptides were registered in subjets whose Abs positivity was at first incomplete (n = 8), with the exception of two youth whose Abs status remained unvaried. Eight individuals lost their immune reactivity in the course of 1–3 years but not before the age of 5. These observations are reflected by changes in responsiveness over the time-points registering decreasing trends in prevalence of Abs against MAP/ZnT8 homologs and increased reactivity to MAP/PI, especially MAP2404c_70–85_ (Fig. 2A).

**Figure 3:**
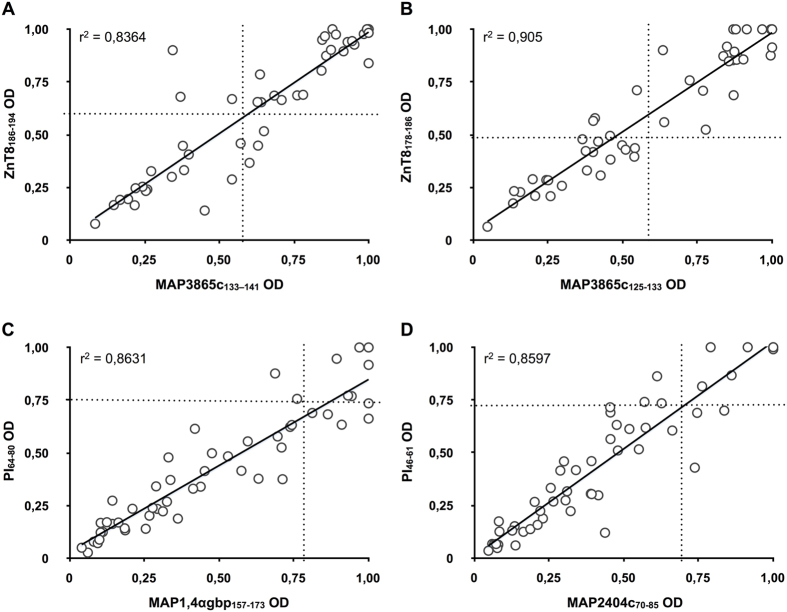
Correlation between Abs recognizing MAP and its homologous human epitopes in Italian children at risk for T1D. Correlations are shown between Abs against (**A**) MAP3865c_133–141_/ZnT8_186–194_, (**B**) MAP3865c_125–133_/ZnT8_178–186_, (**C**) MAP1,4αgbp_157–173_/PI_64–80_ and (**D**) MAP2404c_70–85_/PI_46–61_. Each circle represents Abs detected in one sample. The dotted lines indicate cut-off points for positivity used in each assay, as calculated by ROC analysis.

The overlap between the homologous peptides in all samples of T1D at-risk subjects equaled 83,87% for MAP3865c_133–141_/ZnT8_186–194_, 73,33% for MAP3865c_125–133_/ZnT8_178–186_, 41,38% for MAP1,4αgbp_157–173_/PI_64–80_ and 55,88% for MAP2404c_70–85_/PI_46–61_. Among HCs, this pairwise reactivity was complete for MAP3865c_125–133_/ZnT8_178–186_ followed by 50% for MAP3865c_133–141_/ZnT8_186–194_ and MAP1,4αgbp_157–173_/PI_64–80_, and reaching 40% of overlap for MAP2404c_70–85_/PI_46–61_. A high degree of correlation (r^2^ > 0.8) was found between anti-MAP and the respective anti-ZnT8/PI Abs titers with an even greater coefficient value (r^2^ > 0.9) for the MAP3865c_125–133_/ZnT8_178–186_ pair (Fig. 3).

**Figure 4:**
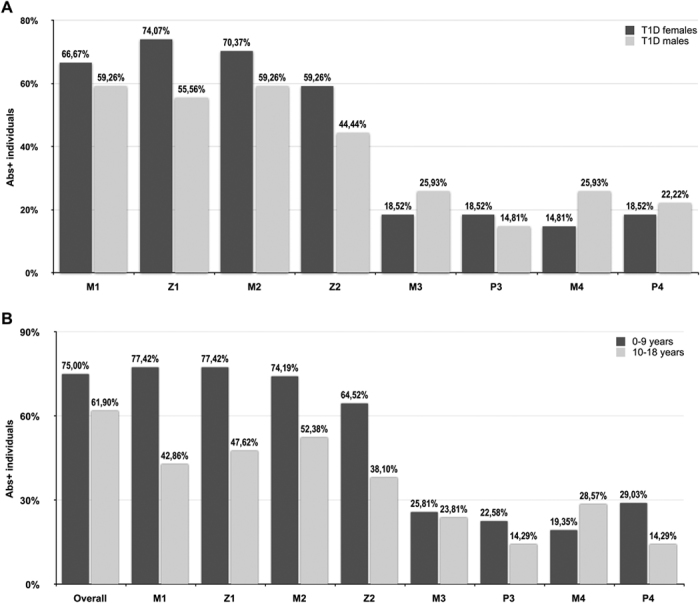
Age/sex-related prevalence of Abs against MAP, proinsulin and ZnT8 homologous epitopes in T1D at-risk subjects. Percentage of children with Abs positivity to selected epitopes upon single-peptide analysis: MAP3865c_133–141_ (M1); ZnT8_186–194_ (Z1); MAP3865c_125–133_ (M2); ZnT8_178–186_ (Z2), MAP1,4αgbp_157–173_ (M3); PI_64–80_ (P3); MAP2404c_70–85_ (M4); PI_46–61_ (P4). (**A**) Sex-related Abs status of positive individuals is indicated by dark grey bars for females and light grey bars for males. (**B**) Analysis performed for 0–9 (dark grey bars) and 10–18 (light grey bars) year-old groups. The first column pair indicates reactivity to any of the analysed peptides.

Upon sex-related analysis of at-risk children, females presented a higher prevalence of Abs directed against MAP/ZnT8 (59,26–74,07%; p < 0.05), with a greater positivity obtained for ZnT8_186–194_ and MAP3865c_125–133_ epitopes (Fig. 4A). Prevalence to the same peptide pairs in males (44,44–59,26%) reached the highest values for both MAP-derived peptides homologous to ZnT8. These trends, even though with lower percentages (14,81–18,25% vs. 14,81–25,93%), were inverse for MAP/PI homologs in both genders with the exception of PI_64–80_ for which females displayed a slightly higher prevalence.

Samples were further analyzed after grouping in two age ranges (0–9 and 10–18 years) showing 75% of prevalence to any of the analyzed epitopes in the younger group when compared to children older than 9 years for whom 61,9% of positivity was registered (AUC = 0.56; p < 0.04; Fig. 4B). However, a complete serum reactivity to the 8 peptides was found throughout different ages in both groups. Upon single-peptide analysis, the highest positivity was obtained at equal levels for MAP3865c_133–141_/ZnT8_186–194_ homolgs (71,88%) within 0–9 years, while the lowest response in the same group corresponded to MAP2404c_70–85_ (15,63%). The 10–18 year-old children displayed the highest prevalence for MAP3865c_125–133_ (52,38%), whereas Abs against both proinsulin epitopes were less recognized (14,29%).

### Prevalence of anti-MAP/ZnT8 and anti-MAP/PI Abs in correlation with positivity to classical islet autoantibodies and the onset of autoimmune diabetes in subjects at risk for T1D

**Figure 5:**
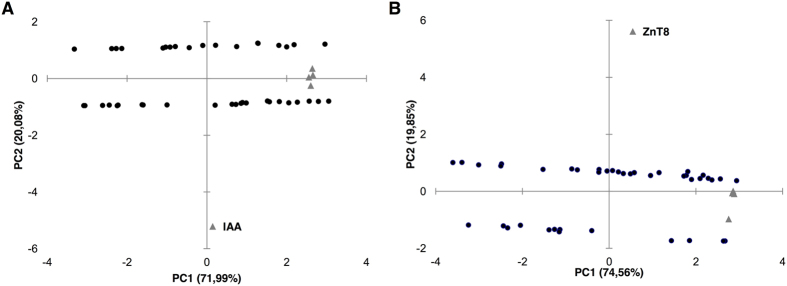
Principal component analysis of MAP/ZnT8 and MAP/PI epitopes with classical islet autoantibodies in T1D at-risk and new onset samples. Bi-plots show correlation between the analyzed homologous peptides and classical antigens as autoimmunity variables: (**A**) anti-MAP/PI Abs and IAA; (**B**) anti-MAP/ZnT8 and classical ZnT8 Abs. Samples are represented by circles whereas triangles indicate variables; triangles without labels illustrate the respective MAP peptides and their human homologs. In both cases samples positive and negative to the corresponding classical islet autoantibodies are distributed in two separated sets along X axis.

Screening for classical islet autoantibodies in the first time-point samples revealed that ZnT8 were the most frequent Abs detected in 58,82% of at-risk subjects for which Abs status was determined, followed by IA2A (42,10%), GADA (40%) and IAA (28,95%; Table 1). This picture changed following to further time-related analyses available for 13 subjects (Fig. 2B). Among at-risk children with all measurements available, three (8,57%) resulted negative to both classical islet autoantibodies and the homologous peptides; one of them was affected by coeliac disease with impaired glucose tolerance and developed high IAA levels at the second blood collection. Principal component analysis indicated a correlation between MAP/ZnT8 and MAP/PI homologs with classical islet autoantibodies (Fig. 5) showing a clear separation of samples positive and negative to ZnT8 or IAA in two horizontal sets. Interestingly, most samples positive to the analyzed MAP/ZnT8 epitopes had also anti-ZnT8 Abs; similar but less pronounced trends were visible between MAP/PI homologs and IAA.

Four at-risk children (13,79%) developed diabetes at the age of 6–11 years old with variable Abs status. One child was positive to IAA, IA2A and ZnT8, the latter exceeding 1000U/ml at T1D onset (9 years) and a complete reactivity to the 8 analyzed peptides. In contrast, a case with only ZnT8 measurements performed became Abs-negative when diagnosed one year later (11 years); likewise no Abs targeting MAP or the homologous epitopes were detected. Only one child developed a full set of classical islet autoantibodies at T1D onset (6 years) but not responded to the homologous peptides. A complete Abs response detected in another case at the second blood collection reduced to GADA and IA2A two years later at T1D diagnosis (11 years); this trend had a similar final for the investigated epitopes as the Abs pattern initially positive to anti-MAP/ZnT8 Abs (9 years) switched to MAP2404c_70–85_ and PI_46–61_ at the second blood collection one year later and reduced to no response at T1D onset.

Out of five subjects considered at high T1D risk due to their classical Abs status, one displayed initially a complete response to islet autoantibodies; this ratio increased to four upon further time-ponit analyses following fluctuations of Abs levels. Considering children at low or moderate risk for T1D, 42,85% were at first positive to multiple classical islet autoantibodies, while 17,86% subjects presented single Abs reactivity; however, data relative to the measured Abs levels were not available in some cases (Table 1). Further analysis of time-point samples revealed an almost unvaried prevalence of positivity to at least two classical islet autoantibodies (42,31%) while reactivity to single Abs increased twice (34,61%); individuals who lost or maintained their Abs negativity accounted for 23,08%.

Among subjects with a complete Abs identification, 55,56% of children positive to at least two classical autoantibodies reacted to not less than 4 homologous peptides, while no response was registered in 22,22%. On the other hand, individuals negative to classical islet autoantibodies presented the same levels of positivity to the analyzed epitopes. Both percentages equaled 33% in children positive to only one classical autoantibody. Of the six cases with a complete response to the homologous MAP and ZnT8/PI peptides, 16,67% showed a full set of classical autoantibodies, 33% had at least a double positivity and in 50% no response was detected.

### Prevalence of anti-MAP/ZnT8 and anti-MAP/PI Abs in association with risk factors in T1D at-risk subjects

37,78% (n = 17) of at-risk subjects had high-risk HLA genotypes, however only 17,64% (n = 3) of them developed T1D and 58,83% were positive to at least half of the analyzed MAP-derived and ZnT8/PI homologous peptides. Another child who progressed to diabetes had a low risk HLA genotype and reacted to MAP3865c_133–141_/ZnT8_186–194_, MAP3865c_125–133_ and MAP2404c_70–85_/PI_46–61_. Males accounted for 58,82% of at-risk subjects with a high-risk HLA genotype, however the ratio of T1D onset between genders equaled 1:1. 54,05% of subjects with low-risk genotypes responded to at least four epitopes, whereas 50% of individuals with HLA DQA1*0501/DQB1*0201 and DQA1*0201/DQB1*0202 genotypes resulted negative.

When Abs status against the selected epitopes was analyzed in correlation with concomitant diseases in subjects at risk for T1D, 82,35% of children affected by coeliac disease or with coeliac familiarity resulted positive to at least two peptides. All individuals with autoimmune thyroiditis occurring alone or combined with coeliac disease reacted to at least four peptides including both MAP/ZnT8 homologous pairs. Among children with T1D familiarity, positivity to at least two epitopes was detected in 53,84% of cases. The lowest ratio (42,86%) was obtained for subjects suffering from occasional hyperglycemia, impaired glucose tolerance and/or obesity. Progression to T1D was associated with the disease familiarity coupled to a high-risk HLA genotype in two cases and a complete Abs positivity to the homologous peptides in one of them, whereas no Abs response was registered in the other one. Occasional hyperglycemia characterized the other two children at T1D onset, one of which without high genetic risk.

A full set of Abs directed against MAP and the homologous epitopes was observed in three cases with T1D familiarity and three cases with coeliac disease or familiarity, whereas immune responses to the peptides were absent in 38,46% of children with T1D familiarity, 57,14% suffering from occasional hyperglycemia and 11,76% with coeliac disease or familiarity. No correlation with age, high-risk HLA genotype or concomitant diseases was found for any of the classical autoantibodies.

## Discussion

Considering the association of MAP with autoimmue diabetes in adults and new-onset children suggested by our previous studies, we investigated here Abs responses against MAP peptides and their human homologs derived from ZnT8 and proinsulin in Italian children at risk for the disease. The present results are in line with our earlier report involving T1D at-risk Sardinian subjects enrolled in the TRIGR project^14^,^18^. Both studies registered a high serum reactivity to MAP-derived epitopes in comparison with healthy individuals; while Sardinian children responded better to MAP/PI homologs, in the present work a particularly high prevalence was obtained for MAP/ZnT8. The difference may be due to a younger mean age of the Sardinian participants with follow-up not exceeding 10 years old in most cases. This picture changed further upon analysis of time-point plasma samples portraying downward trends of positivity to MAP/ZnT8 homologs and proinsulin peptides, paralleled by an increased response to MAP1,4αgbp_157–173_ and MAP2404c_70–85_.

In contrast to evidences claiming IAA as the first circulating autoantibody produced by subjects genetically susceptible to or affected by T1D and being their levels inversely proportional to age^19^, we detected IAA prevalence increased during follow-up. An opposite trend was followed by Abs against classical ZnT8 epitopes that diminished by almost 35%. Interestingly, levels of anti-MAP/ZnT8 Abs declined as well with further time-related analyses. Prevalence of GADA decreased slightly in consonance with reports describing its appearance pattern in distinct age periods^20^.

A cross-reactivity of the analyzed peptides is emphasized by the overlap between the homologous peptides that exceeds 80% for MAP/ZnT8. The pairwise positivity was lower in case of MAP/PI homologs, probably caused by a gradual loss of immunity with increasing age. Principal component analysis permitted to determine a correlation between the peptides and classical islet autoantbodies indicating the strongest link between variables in two antigen groups: MAP/ZnT8 and classical ZnT8 Abs, and MAP/PI and IAA. Further data analysis showed that among IAA-positive subjects, 45,45% had Abs directed against MAP/PI epitopes; this number equaled 25% in IAA-negative children. Similarly, reactivity to MAP/ZnT8 homologs accounted for 80,95% among individuals positive to classical ZnT8 and reached 66,67% among those without anti-ZnT8 Abs. This difference, however, could be attributed to a possible future implication of autoimmune thyroiditis; we have already reported a high prevalence of anti-MAP/ZnT8 in patients affected by Hashimoto’s thyroiditis^21^. Coincidence of autoimmune diseases, with AITD most frequently complicating T1D, is well known to the scientific literature^22^, therefore positivity to ZnT8-derived homologous peptides may indicate an increased risk for multiple autoimmune syndroms. In fact, all patients suffering from autoimmune thyroiditis were positive to the analyzed peptides. This ratio was still high for coeliac disease (82%) but much lower in case of other concomitant symptoms such as occasional hyperglycemia, impaired glucose tolerance and/or obesity (42%). An estimated familial clustering accounts approximately for 40–50%^23^ and in this study was reflected by immune reactivity to MAP-derived epitopes and their homologs. Moreover, male sex has been considered a T1D risk factor for siblings^24^; an equal ratio of positivity to the homologous peptides in males and females could point at a combined gender-related effect, even though, in contrast to autoimmune thyroiditis concerning prevalently women, boys and girls are equally affected by T1D in young populations^25^.

A much higher female reactivity to MAP/ZnT8 may be predictive of a further AITD onset. Classical anti-ZnT8 Abs along with high GADA titers have been considered a risk biomarker for AITD in LADA patients^26^. Males presented a higher response to MAP-derived homologs of proinsulin in different age periods suggesting the impact of differing immune responses to early environmental exposures. In both genders, Abs against MAP/PI always appeared accompanied by responses to MAP/ZnT8; furthermore, positivity to all four MAP/PI homologs was present only with a complete reactivity to MAP/ZnT8 hinting at a high sensitivity of the former epitopes indicating the asymptomatic phase of prediabetes.

Numerous reports evaluating genetic factors in the development of T1D confirm that HLA-DQ2 and HLA-DQ8 haplotypes strongly predispose to the disease. Yet, heterozygous individuals have increased susceptibility compared to homozygous subjects, in particular the combination of DRB1*03:01-DQA1*05:01-DQB1*02:01 and DRB1*04:01/02/04/05/08-DQA1*03:01-DQB1*03:02/04 (or DQB1*02), shortly denoted as DR3/DR4 genotype, confers the highest risk for T1D^27^,^28^. In this view, 62,22% of our samples had low-risk or protective genotypes, however 3 out of 4 cases who progressed to overt diabetes carried a high-risk HLA genotype. Regardless epidemiological studies indicate <10% of high-risk genotypes progressing to islet autoimmunity, 58% of subjects with HLA-conferred susceptibility presented multiple reactivity to the homologous peptides. A slightly lower prevalence among children with low-risk genotypes confirms the previous association of anti-MAP Abs with HLA DQA1*0201/DQB1*0202 at T1D onset^29^ that, together with HLA DQA1*0501/DQB1*0201, in the present study were the most frequent genotypes and corresponded to 50% of positivity to the homologous epitopes, whereas immune reactivity linked to DQA1*0201/DQB1*02 was displayed by 4 out of 5 children. Furthermore, these genotypes confer the highest risk for coeliac disease in homozygous individuals^30^. A greater number of individuals should be analyzed in order to investigate further the association of MAP with HLA genotypes and hypothesize a possible promotion of molecular mimicry between the selected peptides and islet autoantigens.

More cases positive to MAP/ZnT8 registered among at-risk children and a poor reactivity among HCs are in contrast with prevalence of Abs against MAP/PI homologs presenting a diminished at-risk subject/HCs ratio (3,31:1 for MAP/PI vs. 12,9:1 for MAP/ZnT8); the resulting low statistical significance of data relative to anti-MAP/PI responses might be improved by recruiting a higher number of participants. Possible changes of immune responses against the peptides and an early status of classical Abs should be evaluated at youger age including blood collection at birth, in order to verify whether our findings relative to Sardinian children at risk for T1D may be similar in other populations. At present, we observed much higher responses to all MAP/ZnT8 homologs in children younger than 10 years old; interestingly, both proinsulin epitopes followed a similar trend, whereas more cases positive to Abs against their MAP-derived homologs were detected in the 10–18 years group. These results are particularly important for implementation of the current methods for early prediction of T1D development by application of the analyzed peptides as biomarkers in clinical practices with regard to groups at increased risk.

In conclusion, we demonstrated here that children at risk of T1D have high levels of Abs against MAP-derived epitopes and the homologous fragments of ZnT8 and proinsulin inversely proportional to age. In particular, females and subjects younger than 10 years old presented a strong reactivity to MAP/ZnT8 peptides. At the same time, a high prevalence was registered for individuals suffering from coeliac disease. In line with our previous reports associating MAP to Hashimoto’s thyroiditis, further investigation of immune responses in these concomitant disorders will help in deciphering the role of MAP in the development of autoimmunity while detection of anti-MAP/ZnT8 Abs could help in prediction of multiple autoimmune syndromes. In this context, a follow-up study will permit to verify whether multiple reactivity to the analyzed peptides, in particular an unvaried complete Abs status, indicates prediabetes phase and leads to overt T1D.

## Methods

### Subjects

54 children and youth (n = 27 males and n = 27 females, mean age 9,42 ± 3,84 years) at risk for T1D verified by the presence of disease familiarity with closely related members (parents or siblings) and/or high risk HLA genotype, and reference control samples of age-matched healthy volunteers (HCs; n = 42, mean age 6,94 ± 3,58 years) without known history of autoimmune disorders were enrolled in the present study following to periodical visits at the Tor Vergata University Hospital of Rome, Italy. Diagnosis of T1D onset was performed upon analyses for the presence of classical islet autoantibodies (ZnT8, GADA, IA2A and IAA), as well as levels of glycated haemoglobin, according to the American Diabetes Association criteria^31^. Venous whole blood samples were collected after obtaining written informed consent from a parent or caretaker for all study participants and separated plasma was analysed for the presence of Abs against ZnT8, PI and the homologous MAP-derived peptides. For 27 patients, further time-point collections were performed, giving in total 105 samples. The study protocols were approved by the Bioethical Committees of the University of Sassari and the Tor Vergata University Hospital of Rome, Italy.

Methods were carried out in “accordance” with the approved guidelines.

Detailed patients data are provided in Table 1.

### T1D-related autoantibodies

Levels of Abs specific to the ZnT8 C-terminal region (268–369, 325R or 325 W) were determined in the sera by Protein A-radioimmunoprecipitation assays according to the protocol of Lampasona *et al.*^32^. Abs positivity threshold was set at the 99th percentile of 100 HCs obtaining the cut-off value exceeding 30 U/mL. Inter-assay coefficient of variation (CV) accounted for 14%, whereas an intra-assay CV equaled 11%.

Abs to insulin, GAD_65_, and IA-2 were measured by radioligand assays using commercial kits (CentAK^®^ IAA RT, CentAK^®^ anti-GAD65, and CentAK^®^ anti-IA2, Medipan, Germany) according to the manufacturer’s instruction. Values are expressed in arbitrary units with the respective Abs thresholds of >0.4, >0.9 and >0.75 U/mL.

### Peptides

Peptides MAP3865c_133–141_ (LAANFVVAL), MAP3865c_125–133_ (MIAVALAGL), MAP2404c_70–85_ (RGFVVLPVTRRDVTDV) and MAP1,4αgbp_157–173_ (GTVELLGGPLAHPFQPL) along with their respective homologous peptides ZnT8_186–194_ (VAANIVLTV), ZnT8_178–186_ (MIIVSSCAV), PI_46–61_ (RGFFYTPKTRREAEDL) and PI_64–80_ (GQVELGGGPGAGSLQPL) were synthesized at >85% purity (LifeTein, South Plainfield, NJ 07080, USA) assessed by HPLC.

### ELISA

Indirect enzyme-linked immunosorbent assays to detect Abs specific for MAP3865c/ZnT8, MAP1,4αgbp/PI and MAP2404c/PI homologous peptide pairs in plasma samples were performed as described elsewhere^13^. Data was normalized to a highly positive control serum included in all assays with Abs reactivity set at 1.0 arbitrary units (U/ml). Optimal cut-off points to discriminate between positive and negative samples were identified based on the receiver operating characteristic (ROC) curves giving 0.49–0.62 U/ml for MAP/ZnT8 and 0.68–0.78 U/ml for MAP/PI peptide pairs with specificity set at 95,24% and 92,86%, respectively. Statistical significance of the data was determined through the Mann-Whitney U test (95% CI) for not normally distributed values or the student’s t-test using Graphpad Prism Version 6.02 software. Principal component analysis was performed using XLSTAT software.

## Additional Information

**How to cite this article**: Niegowska, M. *et al.* Type 1 Diabetes at-risk children highly recognize *Mycobacterium avium* subspecies *paratuberculosis* epitopes homologous to human Znt8 and Proinsulin. *Sci. Rep.* **6**, 22266; doi: 10.1038/srep22266 (2016).

## Figures and Tables

**Table 1:** Demographic and clinical characteristics of T1D at-risk subjects.

ID	Age^a^	Gender^b^	HLA genotype	Risk factor	ZnT8^c^	IAA^d^	GADA^e^	IA2A^f^
R01	12,65	M	DQ2 (DQA1*0201-DQB1*0202) + DQ8	coeliac disease familiarity (brother)	0,00	0,1	0,25	0,11
R02	9,22	F	DQ2 (DQA1*0201-DQB1*02)	coeliac disease	**77,99**	0,00	**1,13**	0,00
R03	4,08	F	DQ2 (DQA1*0201-DQB1*02)	coeliac disease	0,00	**0,51**	**4,10**	**0,90**
R04	5,61	M	DQ2 (DQA1*0501-DQB1*0201)	T1D familiarity (brother)	**45,50**	–	–	–
R05	18,89	M	DQ2 (DQA1*0201-DQB1*0202)	occasional hyperglycemia	**30,00**	0,00	0,00	-
R06	15,18	F	DQ2 (DQA1*0501-DQB1*0201)	T1D familiarity (brother)	3,00	0,3	0,47	0,23
R07	4,94	F	DR4 + DQ8	T1D familiarity (brother)	0,00	**1,52**	**22,00**	**13,80**
R08	8,61	F	DQ2 (DQA1*0201-DQB1*02)	T1D familiarity (brother)	**56,80**	–	–	–
R09	4,91	M	DQ2 (DQA1*0201-DQB1*02)	T1D familiarity (brother)	**69,86**	–	-	–
R10	4,76	F	DQ2 (DQA1*0501-DQB1*0201)	T1D familiarity (brother)	0,00	0,03	0,16	0,27
R11	9,71	F	–	coeliac disease	0,00	0,1	0,09	**1,56**
R12	8,15	M	low risk	T1D familiarity (brother)	**39,40**	0,4	0,25	0,44
R13	5,31	M	–	coeliac disease	0,00	–	–	–
R14	5,24	M	DQ2 (DQA1*0501-DQB1*0201) + DQ8	coeliac disease, autoimmune thyroiditis	**39,99**	**5,98**	**54,70**	0,32
R15	1,92	M	–	suspected coeliac disease	14,00	0,1	0,01	0,03
R16	7,16	F	DR3	occasional hyperglycemia, impaired glucose tolerance	50,30	0,00	0,00	**1,00**
R18	7,03	M	DQ8 (DQB1*0302)	T1D familiarity (brother)	0,00	–	–	–
R19	13,16	F	–	coeliac disease, occasional hyperglycemia	0,00	–	–	–
R20	8,33	M	–	coeliac disease	**92,33**	**2,80**	0,31	0,11
R21	6,50	M	DQ2 (DQA1*0501-DQB1*0201)	T1D familiarity (brother)	14,22	0,02	0,1	0,21
R22	7,97	M	DQ2 (DQA1*05-DQB1*02)	coeliac disease, hyperthyrotropinemia, occasional hyperglycemia	**46,44**	–	0,00	**1,64**
R23	12,87	M	DQ2 (DQA1*05-DQB1*02)	coeliac disease	0,00	0,04	**1,00**	**0,91**
R24	5,74	M	DQ2 (DQA1*0201-DQB1*0202)	coeliac disease, occasional hyperglycemia	**56,16**	0,02	**1,22**	**1,00**
R25	8,28	F	DQ2 (DQA1*0201-DQB1*0202)	coeliac disease, autoimmune thyroiditis	**93,48**	–	–	–
R26	6,32	F	DQ2	autoimmune thyroiditis	**63,45**	–	4,57	–
R27	7,18	M	DR3 + DR4 + DQ2 (DQA1*0501-DQB1*0201/030503)	T1D familiarity (brother)	**74,30**	**0,52**	**1,16**	0,10
R29	14,65	F	DQ2 (DQA1*0501-DQB1*0201)	coeliac disease, vitiligo and autoimmune thyroiditis familiarity	**93,54**	–	–	–
R30	–	F	–	T1D familiarity (brother)	**30,00**	–	–	–
R31	12,92	M	low risk	T1D familiarity (brother)	**47,62**	0,02	0,25	0,53
R32	8,03	F	DQ2 (DQA1*0501-DQB1*0201)	T1D familiarity (brother)	8,00	0,09	**4,93**	0,23
R33	15,12	M	DR3 + DQ2 (DQA1*0501-DQB1*0201)	T1D familiarity (brother)	22,36	–	–	–
R34	5,09	F	DQ2 (DQA1*0501-DQB1*0201) + DQ8	coeliac disease	**33,81**	**8,66**	**33,77**	**13,51**
R35	8,12	F	DQ2 (DQA1*05- DQB1*02) + DR3 + DR4	coeliac disease	0,00	**1,10**	**39,10**	**7,00**
R36	3,97	M	DQ8	T1D familiarity (brother)	**30,00**	–	–	–
R37	12,11	M	low risk	T1D familiarity (brother)	0,00	0,00	0,00	0,00
R38	7,83	F	DQ2 (DQA1*0201-DQB1*0202)	occasional hyperglycemia	**63,36**	0,00	0,00	0,00
R40	11,83	F	DQ2 (DQA1*0501/0201-DQB1*0202/0201)	T1D familiarity (brother)	**71,39**	**9,13**	**18,90**	**19,05**
R41	17,87	M	–	coeliac disease	**30,00**	–	–	–
R43	10,40	M	DQ8 (DQA1*0201-DQB1*0302)	T1D familiarity (brother)	–	0,00	0,00	0,00
R45	14,17	F	low risk	occasional hyperglycemia	**30,00**	0,00	**2,00**	**0,80**
R46	13,48	M	–	occasional hyperglycemia, obesity, T2D familiarity	**34,00**	0,00	0,00	**0,88**
R47	4,82	F	DR7/DR4 + DQ8 (DQA1*0501-DQB1*030503)	T1D familiarity (brother)	86,14	0,00	0,00	0,00
R48	11,86	F	DQ2	T1D familiarity (brother)	30,00	0,00	**1.132,00**	0,00
R49	11,00	F	DQ2 (DQA1*0201-DQB1*02)	coeliac disease, impaired glucose tolerance	0,00	0,00	0,00	0,00
R50	14,15	M	DQ8	T1D familiarity (brother)	0,00	0,00	0,00	0,00
R52	9,12	M	DQ2/DQ8	T1D familiarity (brother)	**1.532,05**	0,90	0,33	**1,00**
R53	10,02	F	DQ2 (DQA1*05-DQB1*02) + DQ8 (DQB1*0302)	TAG, occasional hyperglycemia	41,00	0,00	0,00	0,00
R54	14,06	F	DQ2	T1D familiarity (father), coeliac and Grave’s disease familiarity (sister)	**150,75**	**1,82**	**3,12**	**1,70**
R55	11,41	F	DQ2/DQ8	T1D familiarity (brother)	0,00	0,20	0,52	**1,20**
R56	9,84	M	DQ8	coeliac disease	0,00	9,13	**3,46**	0,16
R57	9,99	M	DQ2	occasional hyperglycemia	**30,50**	0,14	**23,02**	**4,73**
R58	7,36	F	DQ2	T1D familiarity (brother)	0,00	0,00	0,29	0,18
R59	13,64	M	DQ2 (DQA1*05-DQB1*02) + DQ8 (DQB1*0302)	T1D familiarity (brother)	–	0,00	0,00	0,00
R60	5,75	F	DQ2 (DQA1*05-DQB1*02) + DQ8 (DQB1*0302)	T1D familiarity (brother)	–	0,00	0,00	0,00

ID of subjects who progressed to T1D are highlighted by bold characters; subjects with a high T1D risk conferred by the classical Abs status developed upon follow-up analyses are marked by italics. Age and Abs levels are representative for the initial blood collection when multiple time-point samples were available.

^a^Age at blood collection.

^b^F: females, M: males.

^c^Positive when >30 U/mL.

^d^Positive when >0.4 U/mL.

^e^Positive when >0.9 U/mL.

^f^Positive when >0.75 U/mL. Positive Abs values are shown in bold. Hyphens indicate no measurements performed.
